# Emergence of COVID-19 and Patterns of Early Transmission in an Appalachian Sub-Region

**DOI:** 10.13023/jah.0303.02

**Published:** 2021-07-25

**Authors:** Abbey K. Mann, T. Andrew Joyner, Ingrid Luffman, Megan Quinn, William Tollefson, Ashley D. Frazier

**Affiliations:** Quillen College of Medicine, East Tennessee State University; College of Arts and Sciences, East Tennessee State University; College of Arts and Sciences, East Tennessee State University; College of Public Health, East Tennessee State University; College of Arts and Sciences, East Tennessee State University; Division of Natural Sciences, Walter State Community College

**Keywords:** Appalachia, COVID-19, rural health, geospatial analysis, GIS dashboard, outbreak

## Abstract

**Background:**

In mid-March 2020, very few cases of COVID-19 had been confirmed in the Central Blue Ridge Region, an area in Appalachia that includes 47 jurisdictions across northeast Tennessee, western North Carolina, and southwest Virginia. Authors described the emergence of cases and outbreaks in the region between March 18 and June 11, 2020.

**Methods:**

Data were collected from the health department websites of Tennessee, North Carolina, and Virginia beginning in mid-March for an ongoing set of COVID-19 monitoring projects, including a newsletter for local healthcare providers and a Geographic Information Systems (GIS) dashboard. In Fall 2020, using these databases, authors conducted descriptive and geospatial cluster analyses to examine case incidence and fatalities over space and time.

**Results:**

In the Central Blue Ridge Region, there were 4432 cases on June 11, or 163.22 cases per 100,000 residents in the region. Multiple days during which a particularly high number of cases were identified in the region were connected to outbreaks reported by local news outlets and health departments. Most of these outbreaks were linked to congregate settings such as schools, long-term care facilities, and food processing facilities.

**Implications:**

By examining data available in a largely rural region that includes jurisdictions across three states, authors were able to describe and disseminate information about COVID-19 case incidence and fatalities and identify acute and prolonged local outbreaks. Continuing to follow, interpret, and report accurate and timely COVID-19 case data in regions like this one is vital to residents, businesses, healthcare providers, and policymakers.

## INTRODUCTION

In December 2019, the first known cases of SARS-CoV-2, the novel coronavirus that causes COVID-19, were identified in Wuhan, China.^1^ The first known case of SARS-CoV-2 in the U.S. was detected on January 20, 2020, in Snohomish County WA.^2^ On March 11, 2020, the World Health Organization (WHO) announced that the spread of this disease fit the definition of a pandemic,^3^ marking the first time this has happened since the H1N1 pandemic in 2009.^3^

Geographic analyses of COVID-19 have been categorized into five themes^4^: spatiotemporal analysis^5^,^6^ environmental analysis^7^,^8^ data mining,^9^,^10^ web mapping,^11^ and health geography.^12^,^13^ These broad themes display the breadth of tools that have been applied to manage the pandemic at global, national, and provincial level scales, with a call to increase local-scale spatial analyses.^14^

While the number of COVID-19 cases in rural areas during this phase of the pandemic was lower relative to urban areas, the risk of complications and death was higher in rural areas due to the higher proportion of residents >65 years and higher rate of comorbidities such as diabetes, respiratory disease, and obesity.^15^,^16^ Moreover, rural hospitals are challenged by limitations in personnel, equipment, and intensive care unit (ICU) capacity. Along with limited public transportation, these conditions increase the impact of COVID-19 over the long-term in rural spaces.^15^,^16^

Other factors that increase risks of spreading COVID-19 in rural areas include the types of industries that are predominately found in these areas compared to urban areas such as agriculture and food processing facilities. These settings do not provide the opportunity for physical distancing and often individuals are much closer than the recommended 6-foot distance.^17^

Rural areas that had low prevalence of disease early in the pandemic may have been perceived as lower risk areas, with an associated lack of acceptance of prevention practices.^18^ Due to geography, northeast Tennessee (TN) counties have greater linkages to counties to the north (southwest Virginia (VA)) and east (western North Carolina (NC)) than counties in middle or western TN.^19^ These linkages affect travel for work, education, and healthcare, in that residents of the more rural areas in the region travel to urban areas for services otherwise unavailable.

**Figure 1:**
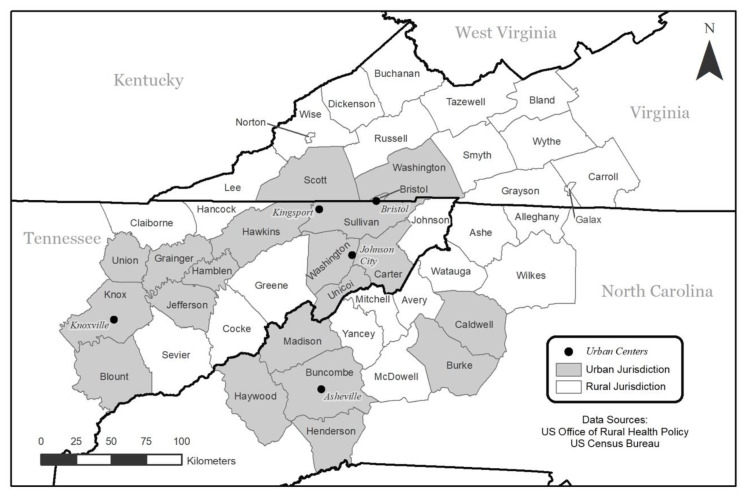
Central Blue Ridge study region reference map with rural/urban region jurisdictions.

The focus of the present study is what is being called the Central Blue Ridge region, which includes parts of Northeast TN, Western NC, and Southwest VA (Figure 1). The region includes 44 counties and three independent cities, hereafter referred to as 47 jurisdictions. Twenty-five of the counties and two of the independent cities included in the region meet the U.S. Office of Rural Health Policy’s definition of rural.^20^ The region also includes five urban areas: Knoxville TN; Asheville NC; Johnson City TN; Kingsport TN; and Bristol TN/VA (Figure 1). The region was initially defined for this research in March 2020 due to its proximity to local healthcare providers in three primary care clinics in northeast TN. The Central Blue Ridge region footprint was refined and expanded by two counties and one independent city in April (2020) based on how COVID-19 data were reported by the health department in VA, resulting in the Central Blue Ridge region as defined in this study.

The goals of the current study were (1) to describe and communicate the emergence of COVID-19 cases in the Central Blue Ridge region using dashboards, graphs, and daily summaries starting with the beginning of state health department reporting in March and continuing through mid-June; (2) to examine acute and prolonged outbreaks during this initial period of shut down and re-opening; and (3) to identify outbreaks at the jurisdictional level using spatial smoothing and clustering methods.

## METHODS

### Data Description

Data were released by each state in the Central Blue Ridge region, and the metrics reported varied in type, detail, and spatial aggregation during the initial phases of the pandemic. The Tennessee Department of Health began releasing information about confirmed COVID-19 cases at the county level by the second week of March.^21^ The North Carolina Department of Health and Human Services reported number of confirmed COVID-19 cases on their website beginning March 16th.^22^ Unlike TN and VA, however, NC reported current rather than cumulative hospitalization and only did so at the state level. The Virginia Department of Health provided case counts at the jurisdictional level (this included counties and independent cities) beginning March 25th.^23^

Beginning on March 18th, 2020, the research team entered daily case counts for each jurisdiction into a spreadsheet for the purpose of tracking cases relevant to primary care providers working in 3 family medicine clinics in the region. Reports on these data, accompanied by COVID-19 research briefs, were disseminated to providers and staff, and archived on a website. By mid-April 2020, the working group collaborated to analyze regional data and disseminate information to a wider audience, which included local providers and the general public, while also coordinating with other researchers and health professionals in the region on a response effort. The study period for the research presented here was March 18–June 11, 2020.

### GIS Dashboard Development

Dashboard development began on March 14th when members of the Geoinformatics and Disaster Science (GADS) Lab at East Tennessee State University collaborated with local emergency managers to form a tracking and resources site for the state. The dashboard included county-level maps showing cases and incidence rates initially, along with hospital and nursing home locations. With the increase in available data released by the Tennessee Department of Health, the Tennessee dashboard expanded to include the following county-level maps in addition to cases and incidence rates: active rate; active rate change (day-to-day); fatalities; fatality ratio (deaths/cases); testing rate (per 100k); and percent tests positive. The dashboard also included plots showing, by day, total cases, total recoveries, total fatalities, new cases, new recoveries, total tests, and new tests.

On April 10th, a new regional dashboard was expanded to include the Central Blue Ridge region. This dashboard included the same types of maps and plots as the TN dashboard with one addition, a daily incidence rate change map. This was important to provide a metric for day-to-day changes at the jurisdiction level since recoveries were not reported at this level in VA or NC. Additionally, tests aggregation varied in VA, with tests eventually being provided at the ZIP code level, which is difficult to aggregate to the county/independent city level since ZIP codes do not always follow the same boundaries. Total and new tests from VA were plotted (not mapped) based on ZIP code locations relative to health department regions. The dashboard also included plots showing, by day, total cases, total recoveries, total fatalities, new cases, new recoveries, total tests (TN and VA), and new tests (TN and VA).

### Statistical and Spatial Analyses

Differences in county-level data reporting among the three states, as noted above, limited Central Blue Ridge regional statistical analyses to statistics that could be constructed from daily cases and daily fatalities, which were reported by each state. To better understand large increases in incidence rates (daily new cases per 100K), local news media were used to create a database of known outbreaks, as state health departments did not report this information. A timeline of known outbreaks was then developed and qualitatively compared to incidence rates and policy decisions (i.e., state closure orders).

**Figure 2:**
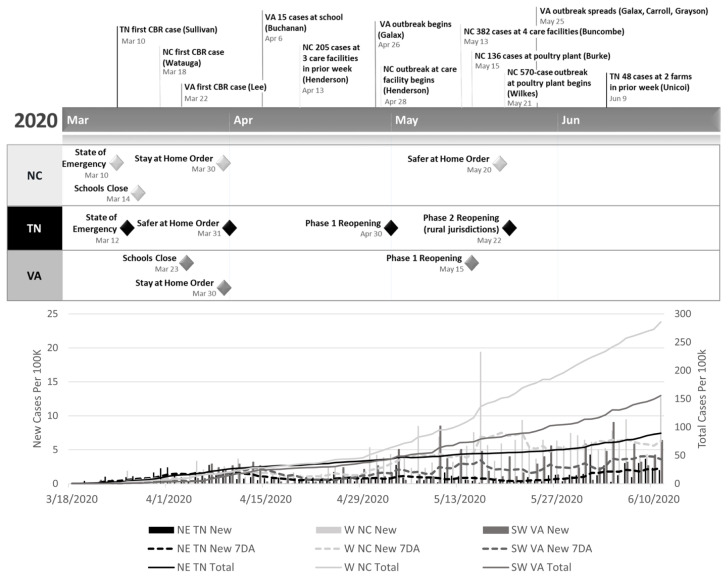
Timeline of outbreaks and government closure and reopening orders in the Central Blue Ridge region, March 10–June 11, 2020, coincides with cumulative total and new COVID-19 Cases per 100K people reported by state health departments

Day-to-day new cases and fatalities in the region were highly variable, reflecting patterns of testing and reporting rather than burden of disease (Figure 2). To mitigate, data were smoothed using the 7-day moving average (7DA). The 7DA of incidence rate, incidence rate change, and fatality rate (COVID-19 deaths per 100K) were joined to a Central Blue Ridge region shapefile in ArcGIS Pro Version 2.4.3 and imported into GeoDa Version 1.14^24^ for cluster analyses. To define the neighborhood for each jurisdiction polygon, a spatial weights matrix was created in GeoDa using five nearest neighbors. The Local Moran’s I statistic^25^ was used to identify high–high and low–low clusters (hot spots and cold spots, respectively) for 7DA incidence rate, incidence rate change, and fatality rate on a weekly time step. Empirical Bayes smoothing using population was employed in the Local Moran’s I calculation to account for spatial variability in variance of the 7DA rates.^26^ Patterns of hot and cold spots were compared for incidence rate, incidence rate change, and fatality rate, week by week.

## RESULTS

### COVID Cases and Outbreaks in the Central Blue Ridge Region

According to the Vintage 2019 county population estimate data,^27^ the region includes a total of 2,715,327 people, 53% (1,444,595) of whom live in northeast TN; 33% (894,618) in western NC; and 14% (376,114) in southwest VA. When data collection began on March 18th, there were already seven confirmed positive cases of coronavirus in the region: one in Watauga County, NC, and six across five counties in northeast TN. By June 11th, there were 4432 total known cases reported in the 47 jurisdictions in the Central Blue Ridge region, equivalent to 163.22 cases per 100,000 residents. Looking at the state level, there were 285.71 cases per 100K in the NC jurisdictions, 155.80 per 100K in the VA jurisdictions, and 89.30 cases per 100K in the TN jurisdictions (see dashed lines in Figure 2).

As of June 11th, in the Central Blue Ridge region, the highest smoothed 7DA incidence rate was 7.5 per 100K occurring on May 19th (NC). Daily incidence rates exceeded the 7DA in all three states at certain times during the study period (May 10th and June 4th (VA); May 16th and June 11th (NC); June 1st (TN), coinciding with outbreaks identified in the timeline (Figure 2).

### Central Blue Ridge Cluster Analyses

**Figure 3:**
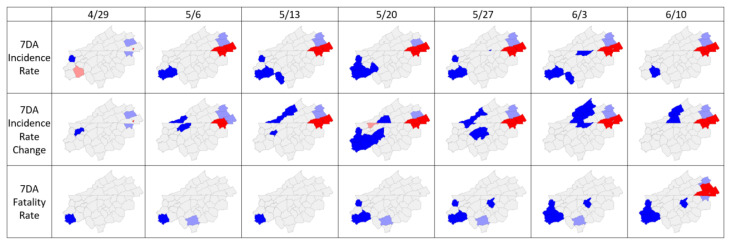
Spatial clusters of COVID-19 7-day moving average (7DA) with Empirical Bayes smoothing by population. Dark red and blue jurisdictions show hot spot and cold spot cluster centers, respectively. Light red shading denotes high values near low values and light blue shading denotes low values near high values, indicating negative spatial autocorrelation.

Empirical Bayes-smoothed COVID-19 incidence rates show persistent hot spots in the eastern portion of the Central Blue Ridge region, specifically in VA (city of Galax and Carroll and Grayson Counties) and NC (Allegheny County) (Figure 3). Note that these jurisdictions represent cluster centers, and the cluster generally extends to neighboring jurisdictions; Wilkes County NC, with a high case count and incidence rate in mid-May was included in the Allegheny County NC cluster as a neighboring jurisdiction. Incidence rate change is useful to show positive increases in incidence rate, indicating increasing rates of infection in these same jurisdictions. Fatality rate shows a delayed response, with a hot spot appearing in city of Galax and Carroll, Grayson, and Wythe Counties in VA 5 weeks after the appearance of the incidence rate hot spot in this part of the Central Blue Ridge region.

### The Central Blue Ridge Region Dashboard as a Communication Tool

**Figure 4:**
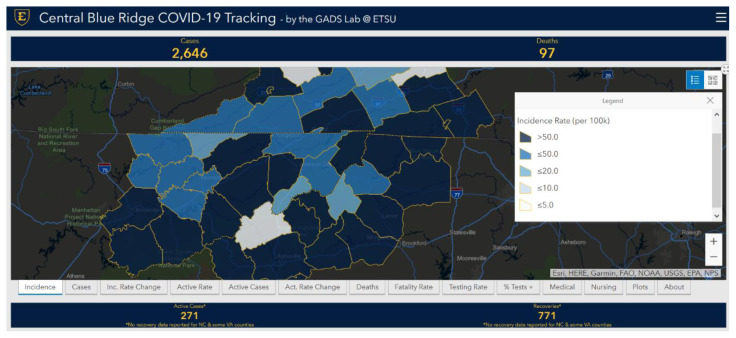
Snapshot of the Central Blue Ridge region dashboard on May 22 with the incidence rate map selected.

The Central Blue Ridge region dashboard became a powerful tool for disseminating information, not only to emergency managers and local officials but also to the general public. The dashboard can be accessed here: http://arcg.is/1fHLjq. Figure 4 shows the incidence rate map for the Central Blue Ridge region on May 22nd. On that day, incidence rates were higher than 50.0 (per 100k) in 11 of 14 NC counties, 8 of 16 VA counties/cities, and 8 of 17 TN counties. Legends indicate sequential and divergent color-coded values.

## DISCUSSION

In March 2020, researchers began documenting the spread of COVID-19 in the Central Blue Ridge region, a somewhat rural area that includes 44 counties and three independent cities in northeast TN, western NC, and southwest VA. While this is not a traditionally defined region, Nelson and Rae^9^ identified a similar area based on “economic geography” indicators, simply calling it “Blue Ridge.”

The basis for this area as a stand-alone region is the result of inter-linked economic and cultural factors, inferring that there is a high degree of communication and commerce within the Central Blue Ridge region. Unlike more densely populated parts of the world, very few cases of COVID-19 had been detected in this region when WHO officially classified the spread of COVID-19 as a pandemic. In the weeks that followed that announcement, schools and businesses in the region shut down and transitioned to remote working, and cities and states urged residents to stay home whenever possible. This analysis shows the initially low number of cases and very limited spread of COVID-19 in much of this region.

Most daily increases in the number of COVID-19 cases in Central Blue Ridge localities were associated with outbreaks in congregate settings (Figure 2). These included outbreaks at a school (Buchanan County VA, April 6); multiple long-term care facilities (Henderson and Buncombe Counties NC, beginning April 4th and May 13th, respectively); two meat-processing facilities (Burke and Wilkes Counties NC, May 15th and May 21st, respectively); and two farms (Unicoi County TN, June 1st and 9th).

In two jurisdictions, daily increases in cases per 100K were associated with widespread community transmission. First, the city of Galax VA experienced an outbreak that began with family gatherings and spread to the community via local workplaces.^28^ This prolonged outbreak emerged as a Galax–Carroll–Grayson incidence rate hot spot in the cluster analyses (Figure 3) the week of May 6^th^, and as a fatality rate hot spot the week of June 10th (a 34-day lag). This lag between incidence and fatalities agrees most closely with a 28-day lag (onset to death) reported by Yang et al.,^29^ but is over twice the lag of 13 days used in other studies to predict fatality rates.^30^–^32^

The second locality where increasing cases per 100K was associated with community transmission was Burke County NC from June 6 to June 11. Earlier in May, daily case increases were attributed to an outbreak at a poultry plant (Figure 2); however, the June 6–11 increase in new cases was attributed to traveling, congregate living, and community spread.^33^ This suggests that a transition toward community spread may have occurred as the number of cases increased in the region.

Hot-spot analyses failed to identify short-term outbreaks associated with congregate settings outlined in Figure 2. This was likely due to the use of the 7DA as a smoothing function. The use of the 7DA helped to identify prolonged outbreaks. However, due to 7DA smoothing, outbreaks with a high number of cases reported on a single day of testing were unlikely to be identified as statistically significant hot spots. Further, the use of rates (new cases or fatalities per 100K) helped to identify hot spots in smaller, low-population jurisdictions at the expense of larger jurisdictions. For example, outbreaks at long-term care facilities in Henderson County NC were not identified as hot spots because the high county population reduced the incidence rate relative to lower population jurisdictions. Therefore, the cluster-analysis methods employed here are most effective for identification of statistically significant hot spots of incidence or fatality rates in low population localities with relatively few cases and/or areas of persistent and increasing community spread.

Two important limitations of the current study are both inherent to the methods used to identify and report cases of COVID-19. First, lack of widespread access to testing early in the pandemic likely resulted in an underreporting of early case rates. In addition, lack of mandatory testing in most of the region throughout the period examined for this study means detecting cases was dependent on community members voluntarily getting tested and also likely resulted in underestimation of case totals.

### Future Research

Analyses for this study ended on June 11th, but cases continued to increase throughout the summer, especially in the TN counties of the Central Blue Ridge region. Additional research should examine clusters, trends, and policy/event impacts across TN, with unique analysis opportunities based on statewide data homogeneity from the Tennessee Department of Health. Demographic and community-level analysis may also be explored across TN, including analysis of disparate policies implemented at the local level.

## CONCLUSION

This research describes the emergence and progression of COVID-19 cases in eastern TN, western NC, and southwest VA and the efforts to communicate information to the general public, healthcare providers, and officials. Briefs that comprise timely and accurate descriptions of COVID-19 cases in the Central Blue Ridge region are an important mechanism for dissemination of pertinent information about the pandemic, particularly to healthcare providers and staff in the region who are focused on addressing important new health concerns in local communities. Similarly, the GIS dashboard is a powerful tool to communicate spatial data including incidence rate, fatality rate, testing rate, as well as location of hospitals and testing centers.

Regional cluster analyses of smoothed incidence and fatality rates successfully identified statistically significant hotspots (outbreaks) that persisted over a prolonged period. The methods used here were unable to identify outbreaks in which the majority of cases were reported on a single day, due to the use of 7DA. These types of outbreaks occurred when new cases appeared suddenly in congregate settings such as long-term care facilities, meat processing facilities, and farms. This research demonstrates solutions to the challenges of consolidating disparate data from multiple state and regional health departments into useful information for the general public, healthcare providers, and policymakers. It may be advisable for similar geographic regions that include rural and urban jurisdictions in multiple states across which residents frequently travel for work, school, or recreation, to take a similar approach to examining and reporting data. This would improve communication and transparency, while reducing confusion during an event such as a pandemic when new and disparate information can quickly become overwhelming.

**SUMMARY BOX**

**What is already known on this topic?** The study of the process of gathering and sharing information about the emergence and spread of COVID-19 is an emerging area of research.

**What is added by this report?** This report presents a model for tracking and disseminating information about the spread of COVID-19 in a mostly-rural area that spans three states, in which baseline access to care is relatively low.

**What are the implications for future research?** This study lays the groundwork both for the continued tracking of COVID-19 and of similar viruses that involve collection of data from multiple sources.

## References

[1] Li Q Guan X Wu P Early transmission dynamics in Wuhan, China, of novel coronavirus–infected pneumonia New Engl J Med 2020 382 13 1199 207 31995857 10.1056/NEJMoa2001316 PMC7121484

[2] Holshue ML DeBolt C Lindquist S First case of 2019 novel coronavirus in the United States New Engl J Med 2020 382 10 929 36 32004427 10.1056/NEJMoa2001191 PMC7092802

[3] World Health Organization Past pandemics [Internet] Copenhagen, Denmark WHO Regional Office for Europe 2020 Available from: https://www.euro.who.int/en/health-topics/communicable-diseases/influenza/pandemic-influenza/past-pandemics

[4] Franch-Pardo I Napoletano BM Rosete-Verges F Billa L Spatial analysis and GIS in the study of COVID-19. A review Sci Total Environ 2020 739 140033 32534320 10.1016/j.scitotenv.2020.140033 PMC7832930

[5] Desjardins MR Hohl A Delmelle EM Rapid surveillance of COVID-19 in the United States using a prospective space-time scan statistic: detecting and evaluating emerging clusters Appl Geogr 2020 118 102202 32287518 10.1016/j.apgeog.2020.102202 PMC7139246

[6] Giuliani D Dickson MM Espa G Santi F Modelling and predicting the spatio-temporal spread of coronavirus disease 2019 (COVID-19) in Italy BMC Infect Dis 2020 20 700 32967639 10.1186/s12879-020-05415-7 PMC7509829

[7] Ma Y Zhao Y Liu J Effects of temperature variation and humidity on the death of COVID-19 in Wuhan, China Sci Total Environ 2020 724 138226 32408453 10.1016/j.scitotenv.2020.138226 PMC7142681

[8] Qi H Xiao S Shi R COVID-19 transmission in mainland China is associated with temperature and humidity: a time-series analysis Sci Total Environ 2020 728 138778 32335405 10.1016/j.scitotenv.2020.138778 PMC7167225

[9] Chen ZL Zhang Q Lu Y Guo ZM Zhang X Zhang WJ Distribution of the COVID-19 epidemic and correlation with population emigration from Wuhan, China Chinese Med J-Peking 2020 133 9 1044 50 10.1097/CM9.0000000000000782 PMC7147281 32118644

[10] Gao S Rao J Kang Y Liang Y Kruse J Mapping county-level mobility pattern changes in the United States in response to COVID-19 SIGSpatial Special 2020 12 1 16 26

[11] Dong E Du H Gardner L An interactive web-based dashboard to track COVID-19 in real time Lancet Infect Dis 2020 20 5 533 4 32087114 10.1016/S1473-3099(20)30120-1 PMC7159018

[12] Lakhani A Introducing the percent, number, availability, and capacity [PNAC] spatial approach to identify priority rural areas requiring targeted health support in light of COVID-19: a commentary and application J Rural Health 2020 37 1 149 52 32277774 10.1111/jrh.12436 PMC7262053

[13] Allcott H Boxell L Conway J Gentzkow M Thaler M Yang DY Polarization and public health: partisan differences in social distancing during the coronavirus pandemic J Public Econ 2020 191 104254 32836504 10.1016/j.jpubeco.2020.104254 PMC7409721

[14] Ahmadi A Fadai Y Shirani M Rahmani F Modeling and forecasting trend of COVID-19 epidemic in Iran until May 13, 2020 Med J Islam Repub Iran 2020 34 27 32617266 10.34171/mjiri.34.27 PMC7320984

[15] Ameh GG Njoku A Inungu J Younis M Rural America and coronavirus epidemic: challenges and solutions Eur J Environ Public Health 2020 4 2 em0040

[16] Cafer A Rosenthal M COVID-19 in the rural south: a perfect storm of disease, health access, and co-morbidity APCRL Policy Briefs 2020 https://egrove.olemiss.edu/apcrl_policybriefs/2

[17] Centers for Disease Control and Prevention Rural communities and COVID [Internet] Atlanta (GA) Centers for Disease Control and Prevention 2020 July 30 [Updated 2021; cited 2020 Aug 3]. Available from: https://www.cdc.gov/coronavirus/2019-ncov/need-extra-precautions/other-at-risk-populations/rural-communities.html

[18] Prusaczyk B Strategies for disseminating and implementing COVID-19 public health prevention practices in rural areas J Rural Health 2021 37 142 4 32246497 10.1111/jrh.12432

[19] Nelson GD Rae A An economic geography of the United States: from commutes to megaregions PLoS ONE 2016 11 11 e0166083 27902707 10.1371/journal.pone.0166083 PMC5130203

[20] Office of Rural Health Policy List of rural counties and designated eligible census tracts in metropolitan counties [Internet] Rockville (MD) Office of Rural Health Policy 2010 [cited 2020 August 3]. Available from: https://www.hrsa.gov/sites/default/files/hrsa/ruralhealth/resources/forhpeligibleareas.pdf

[21] Tennessee Department of Health Epidemiology and Surveillance Data [Internet] Nashville (TN) Tennessee Department of Health 2020 [cited 2020 Jun 11]. Available from https://www.tn.gov/health/cedep/ncov/data/downloadable-datasets.html

[22] North Carolina Department of Health and Human Services NCDHHS COVID-19 Response [Internet] Raleigh (NC) North Carolina Department of Health and Human Services 2020 [cited 2020 Jun 11]. Available from: https://covid19.ncdhhs.gov/

[23] Virginia Department of Health COVID-19 in Virginia [Internet] Richmond (VA) Virginia Department of Health 2020 [cited 2020 Jun 11]. Available from: https://www.vdh.virginia.gov/coronavirus/

[24] Anselin L Syabri I Kho Y GeoDa: an introduction to spatial data analysis Geogr Anal 2006 38 1 5 22

[25] Anselin L Local indicators of spatial association — LISA Geogr Anal 1995 27 2 93 115

[26] Cressie N Smoothing regional maps using Empirical Bayes predictors Geogr Anal 1992 24 1 75 95

[27] US Census Bureau Annual estimates of the resident population of counties: Vintage 2019 U.S. Census Bureau Population Division 2020

[28] Strother M Galax, Carrol County see spike in coronavirus cases WSLS Channel 10 News [Internet] 2020 Jun 16 [cited 2020 Jul 22]. Available from: https://www.wsls.com/news/local/2020/06/15/galax-carroll-county-see-spike-in-coronavirus-cases/

[29] Yang X Yu Y Xu J Clinical course and outcomes of critically ill patients with SARS-CoV-2 pneumonia in Wuhan, China: a single-centered, retrospective, observational study Lancet Resp Med 2020 8 5 475 81 10.1016/S2213-2600(20)30079-5 PMC7102538 32105632

[30] Linton NM Kobayashi T Yang Y Incubation period and other epidemiological characteristics of 2019 novel coronavirus infections with right truncation: a statistical analysis of publicly available case data J Clin Med 2020 9 2 538 10.3390/jcm9020538 PMC7074197 32079150

[31] Russell TW Hellewell J Jarvis CI Estimating the infection and case fatality ratio for coronavirus disease (COVID-19) using age-adjusted data from the outbreak on the Diamond Princess cruise ship, February 2020 Euro Surveill 2020 25 12 2000256 10.2807/1560-7917.ES.2020.25.12.2000256 PMC7118348 32234121

[32] Wilson N Kvalsvig A Barnard LT Baker MG Case-fatality risk estimates for COVID-19 calculated by using a lag time for fatality Emerg Infect Dis 2020 26 6 1339 441 32168463 10.3201/eid2606.200320 PMC7258483

[33] Burke County Burke County Media Briefing 6/12/2020 [Internet] Morganton (NC) Burke County 2020 [cited 2020 Jul22]. Available from: https://www.burkenc.org/CivicAlerts.aspx?AID=345

